# Tracking Newly Released Synaptic Vesicle Proteins at Ribbon Active Zones

**DOI:** 10.1016/j.isci.2019.06.015

**Published:** 2019-06-13

**Authors:** Thirumalini Vaithianathan, Lonnie P. Wollmuth, Diane Henry, David Zenisek, Gary Matthews

**Affiliations:** 1 Department of Neurobiology and Behavior, Stony Brook University, Stony Brook, NY 11794, USA; 2 Center for Nervous System Disorders, Stony Brook University, Stony Brook, NY 11794, USA; 3 Department of Biochemistry & Cell Biology, Stony Brook University, Stony Brook, NY 11794, USA; 4 Department of Ophthalmology, Stony Brook University, Stony Brook, NY 11794, USA; 5 Department of Cellular and Molecular Physiology, Yale University School of Medicine, New Haven, CT 06520-8066, USA; 6 Department of Ophthalmology and Visual Sciences, Yale University School of Medicine, New Haven, CT 06520-8066, USA

**Keywords:** Optical Imaging, Neuroscience, Cellular Neuroscience

## Abstract

Clearance of synaptic vesicle proteins from active zones may be rate limiting for sustained neurotransmission. Issues of clearance are critical at ribbon synapses, which continually release neurotransmitters for prolonged periods of time. We used synaptophysin-pHluorin (SypHy) to visualize protein clearance from active zones in retinal bipolar cell ribbon synapses. Depolarizing voltage steps gave rise to small step-like changes in fluorescence likely indicating release of single SypHy molecules from fused synaptic vesicles near active zones. Temporal and spatial fluorescence profiles of individual responses were highly variable, but ensemble averages were well fit by clearance via free diffusion using Monte Carlo simulations. The rate of fluorescence decay of ensemble averages varied with the time and location of the fusion event, with clearance being most rapid at the onset of a stimulus when release rate is the highest.

## Highlights

• We monitor single-molecule clearance after exocytosis in ribbon synapses of zebrafish
• Diffusion of synaptophysin is dependent on the time and localization of fusion events
• Synaptophysin diffusion is slower than reported in other systems
• Bipolar cell synapses may not be rate limited by protein clearance after exocytosis

## Graphical Abstract

**Figure:**
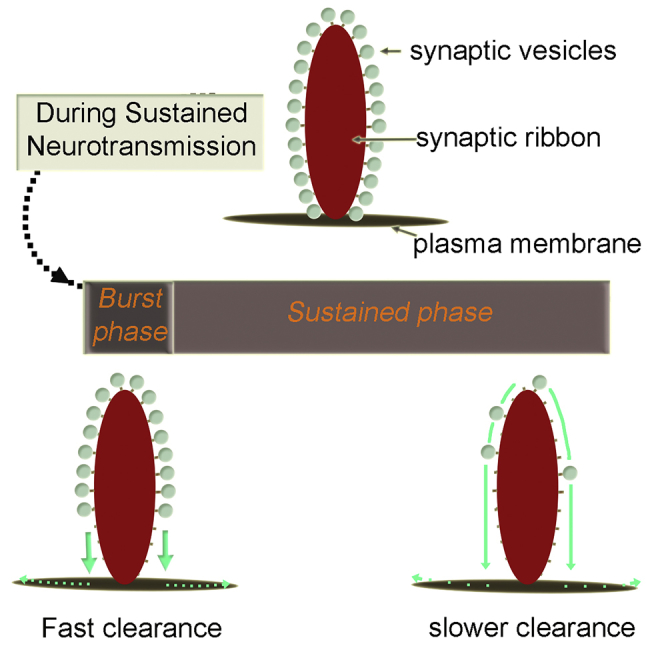

## Introduction

Ribbon synapses of sensory neurons support continuous exocytosis in response to sustained depolarization (Matthews and Fuchs, 2010). To support the continuous release of neurotransmitters, rapid replenishment of release-ready synaptic vesicles at presynaptic active zones is required. At conventional, non-ribbon-type synapses, clearance of fused synaptic vesicle proteins from the release site is an important determinant of recovery from short-term synaptic depression, and the clearance and availability of release sites has been suggested to be rate limiting during sustained synaptic release (Hosoi et al., 2009, Hua et al., 2013, Neher, 2010, Rajappa et al., 2016, Wu et al., 2009). Furthermore, at rod ribbon synapses, maintenance of endocytosis, which is an important mechanism to clear synaptic vesicle proteins, is required to maintain release even at moderate frequencies (Wen et al., 2018). Therefore, clearance of release sites may be of great importance during sustained release at ribbon synapses.

To study protein clearance following exocytosis, we took advantage of a line of zebrafish, which weakly express the exocytosis reporter, SypHy (synaptophysin-pHluorin fusion protein), a transgene driven by the HSP70 promoter (Vaithianathan et al., 2016). Increase in SypHy fluorescence in response to stimulation reports exocytosis, when the interior of the vesicle connects with the external medium (Sankaranarayanan et al., 2000). By using a weakly expressing line, we can monitor the fate of individual molecules following exocytosis with high spatial and temporal precision. To localize fusion of single vesicles and trafficking of synaptophysin after fusion, we imaged vesicle trafficking at the active zone of bipolar cell ribbon synapses at high spatial resolution (Vaithianathan et al., 2016). In this study, imaging of single SypHy molecules using rapid confocal line scans reveals a decay in fluorescence, largely reflecting lateral movement of synaptophysin molecules out of the imaging locale. In addition, our results indicate that when and where a fusion event occurs correlates with the amount of time the synaptophysin molecule resides in the imaging region, with clearance being accelerated at times when exocytosis rates are the highest.

## Results

To track the dynamics of a fused vesicle protein, we used transgenic zebrafish expressing SypHy under control of a heat shock promoter (Vaithianathan et al., 2016). SypHy is essentially nonfluorescent when the fluorophore is contained within the acidic environment of a synaptic vesicle (Balaji and Ryan, 2007, Granseth et al., 2006, Sankaranarayanan et al., 2000, Voglmaier et al., 2006, Wu et al., 2009), but rapidly becomes fluorescent upon exocytosis as it deprotonates. Hence, SypHy is a reliable marker of plasma-membrane synaptophysin localization following exocytosis. We specifically chose low-expressing SypHy transgenic zebrafish for the following reasons: (1) low-expressing cells are less prone to overexpression artifacts, such as saturation of membrane retrieval mechanisms, and thus may more reliably report endogenous protein behavior and (2) overexpression may lead to excess surface expression (Royle et al., 2008), reducing the likelihood of detecting individual molecules at high spatial and temporal resolution.

**Figure 1:**
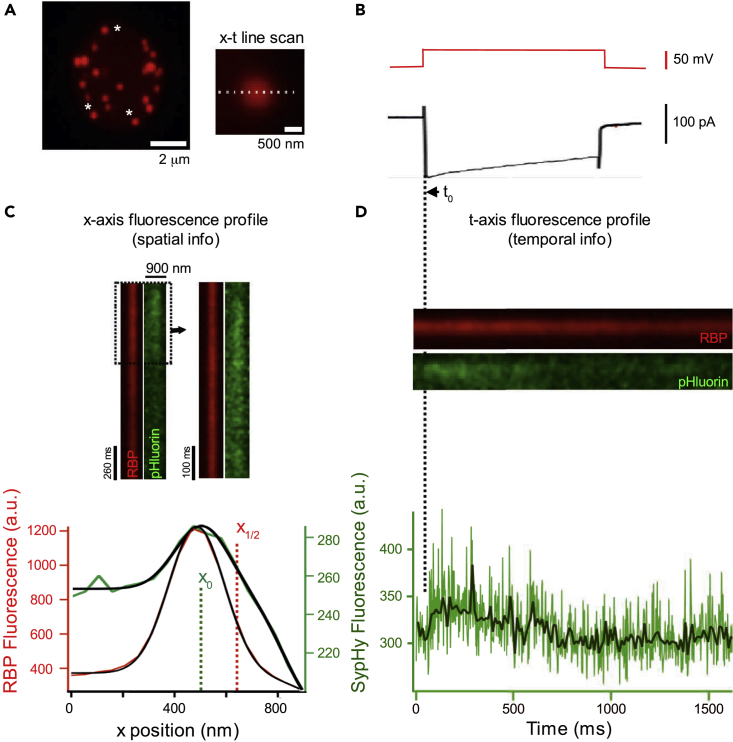
High-Resolution Imaging of SypHy Fluorescence Reports Spatiotemporal Aspects of Synaptic Vesicle Fusion at a Single Ribbon Active Zone (A) Overview of experimental procedure. (Left) 2D projection from a series of confocal optical sections through a zebrafish bipolar cell synaptic terminal. A synaptic terminal was voltage clamped using a whole-cell pipette with an internal solution containing deep-red-fluorescent RBP (CF633-RBP) to label synaptic ribbons. RBP fluorescence was concentrated at ribbons and also filled the entire terminal, which allowed visualization of the terminal border. Experiments were carried out on ribbons that could be distinguished from adjacent ribbons (e.g., white asterisks). (Right) Close-up view of a single synaptic ribbon. The outside of the cell is to the right, which is the standard orientation adopted for all images. x-t scan lines (broken line) were positioned perpendicular to the plasma membrane, extending from the intracellular side of the ribbon to the extracellular space. These x-t raster plots were used to measure the fluorescence intensity profiles of RBP (red) and SypHy (green) shown in (C and D). (B) Voltage-clamp recording of a bipolar cell terminal. Terminals were held at −60 mV and stepped to −10 mV (t_0_) for 1 s (red) to evoke a sustained Ca^2+^ current (black). Logic pulses exchanged between patch-clamp and imaging computers synchronized acquisition of x-t line-scan and imaging data. A typical experiment begins with voltage command (V_H_ = −60 mV); a transistor-transistor logic (TTL) pulse generated from Patch Master triggers image acquisition. t_0_ is the time of depolarization (see Methods). (C) Illustration of the approach to obtain the spatial location of SypHy fluorescence with respect to the ribbon. (Top) Example of an x-t raster plot oriented to illustrate x axis intensity profiles of RBP (red) and SypHy (green) fluorescence during sustained depolarization. (Bottom) Fluorescence intensity profiles along the x axis for RBP (red) and SypHy (green) were fit with a sigmoid Gaussian function (see Methods) (Vaithianathan et al., 2016). The centroid (x axis position) of RBP and SypHy was taken as the peak of the Gaussian fit (x_0_, shown by dotted green line for SypHy). The parameter x_1/2_ (dotted red line) from the sigmoid fit to the RBP fluorescence (red trace) was used to estimate the location of the plasma membrane. (D) (Top) x-t raster plot, same recording as in (C) but reoriented to demonstrate t axis intensity profiles of RBP (red) and SypHy (green) fluorescence during sustained depolarization. Scans were averaged over 5 pixels. (Bottom) Raw (light green) or averaged (black line) temporal intensity profile of the SypHy fluorescence over a region of interest (see also Figure S1). See also Figures S1 and S2.

Retinal bipolar cells contain vesicle-binding scaffolds, known as synaptic ribbons, which localize vesicles to active zones. Synaptic ribbons are made up largely of the protein Ribeye. In this study, synaptic ribbons were labeled with a fluorescent peptide that binds to Ribeye (CF-633-RBP) (Vaithianathan et al., 2016, Zenisek et al., 2004) delivered via a patch pipette placed directly on a bipolar terminal (Figure 1A, left). SypHy fluorescence was monitored by line scan confocal imaging along the length of the synaptic ribbon in a direction perpendicular to the plasma membrane (Figure 1A, right). To evoke exocytosis, the membrane potential was stepped to −10 mV for 1 s from a resting potential of −60 mV (Figure 1B, red line), where we measured both membrane current (Figure 1B) and capacitance (not shown). From these line scans, we derived the spatial (Vaithianathan and Matthews, 2014) (Figure 1C) and temporal (Figure 1D) aspects of SypHy fluorescence relative to the synaptic ribbon and plasma membrane.

### Spatial Aspects of SypHy Fluorescence Report Locus of Synaptic Vesicle Fusion Relative to a Single Ribbon Active Zone and the Plasma Membrane

To determine the locus of vesicle fusion, we analyzed the x axis profiles of SypHy fluorescence relative to a synaptic ribbon and the plasma membrane (Figure 1C) (Vaithianathan et al., 2016). For consistency, we display line scans such that the outside of the cell is to the right. Although SypHy fluorescence was detectable in individual scan lines, the low signal relative to noise precluded accurate localization. We therefore averaged line scans over 2 pixels in the x axis and 5–8 lines in the t-axis (Figure S1 and Methods). The fluorescence intensity profile of SypHy events perpendicular to the membrane was then analyzed to determine the position of SypHy events relative to the ribbon and the plasma membrane (Figure 1C). A histogram of the x axis position of 81 SypHy events (Figure S2) indicates that as reported previously (Vaithianathan et al., 2016) they were broadly spread across the ribbon and were not confined to positions near the plasma membrane.

### Characterization of Individual pHluorin Events

**Figure 2:**
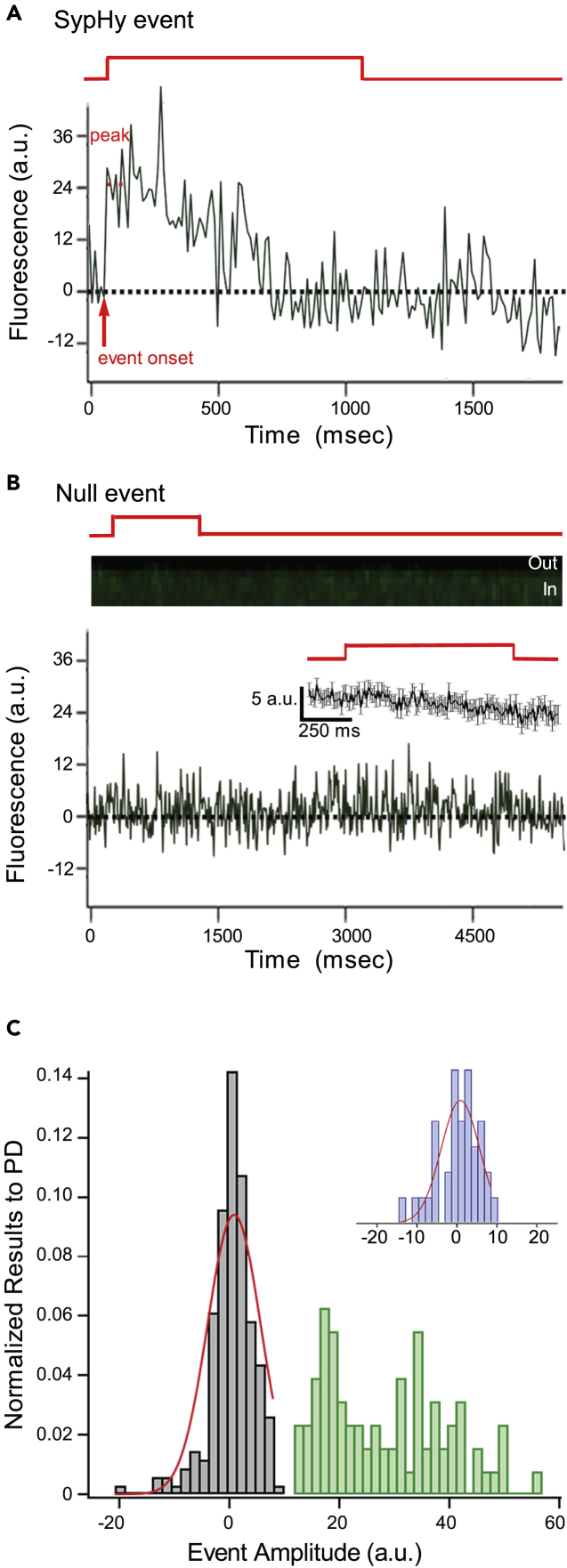
SypHy Fluorescence Reports Single Fusion Events (A and B) Examples of averaged temporal intensity profile (see Figure S1) of green fluorescence illustrating the occurrence of either an SypHy (A) or a null (B) event. For analysis and display, fluorescence was normalized to baseline fluorescence before the depolarization. Detection of SypHy events and analysis of amplitudes were determined as explained in Methods. Briefly, events were visually identified from x-t scans, and only events with ≥1.5 SDs above the average amplitude of baseline noise (see Figure 2C, inset) were defined as SypHy events. Depolarizations that failed to elicit an identifiable event were defined as “null” events (B). (B, inset) Ensemble of 203 randomly chosen null events (see panel C, gray bars). The voltage protocols (red) indicate the timing of the depolarization. For all recordings, including null events, capacitance measurements with sustained depolarization reported strong increases in capacitance (∼150 fF) indicating robust exocytosis. (C) Histogram showing distribution of event amplitudes for null and SypHy events. Amplitudes were measured for 203 randomly selected nulls (out of 1,372 nulls total) (see Methods). The results (gray bars) were normalized to the total number of nulls (probability density). The average amplitude of null and SypHy events were −0.6 ± 0.3, N = 203, and 27.1 ± 1.2, N = 81, respectively. The distribution of null amplitudes is well fit (red line) by the same Gaussian function that defines the noise (i.e., in the absence of a stimulus) (inset) with the amplitude as the only free parameter. (Inset) Histogram of fluorescence changes, ΔF, in the absence of stimulation provides the baseline variability, the imaging noise. Baseline noise fluorescence was obtained using the same approach as with SypHy and nulls, except that noise measurements were made in the absence of a stimulus (see Methods). The results were normalized to the total number of trials and fit with Gaussian function in red (σ = 4.9) (noise average amplitude: −0.4 ± 0.9; N = 36). See also Figures S3 and S4.

Depolarization of bipolar cells occasionally gave rise to events, characterized by rapid increases in fluorescence above baseline followed by a slower decay that we took to represent the exocytosis of a single vesicle (see below). To track the dynamics of such SypHy fusion events, we analyzed SypHy fluorescence in time relative to the stimulating voltage step (Figure 1D). SypHy signals appeared abruptly as expected for the nearly instantaneous loss of vesicle protons upon fusion and persisted for some time before declining to baseline fluorescence (Figure 1D, lower panel) indicating movement of the SypHy molecule out of the x-t line scan, endocytosis followed by reacidification, or photobleaching.

x-t line scans were obtained from 1,453 ribbons during sustained depolarization (Figure 2). We identified SypHy events in 81 of these recordings and characterized their amplitude (e.g., Figure 2A) (see Methods). The majority of line scans (1,372) exhibited no detectable events (e.g., Figure 2B), which we refer to as “null” events. It is possible that there are errors in the distinction between SypHy and null events, but we think these errors are minimal for several reasons: first, we characterized the baseline or imaging noise in the absence of a stimulus (Figure 2C, inset). This distribution was well fit by a Gaussian (red line) with an average amplitude of −0.4 a.u. and an SD of 4.9 (N = 36). The average amplitude of presumed SypHy events (27.1 ± 1.2 a.u., N = 81) (Figure 2C, green bars) far exceeds this noise level with the bulk of the events having amplitudes >1.5 SD. Second, to characterize the amplitude of presumed null recordings, we randomly selected 203 recordings without detectable events and measured the amplitude right after the voltage step (Figure 2C, gray bars). These amplitudes were well fit by a Gaussian function with parameters (average amplitude of −0.6 a.u., SD of 4.0, N = 203) comparable to the imaging noise, consistent with these nulls arising from noise. Third, to test for whether there could be hidden SypHy events in the noise of presumed null events, we averaged the 203 randomly selected events by aligning them to the start of the depolarization, when exocytic rate is the highest (Figure 2B, inset). If we are consistently missing events beneath the noise floor, signal averaging across many traces would reduce the noise and reveal this signal. However, no dramatic changes in fluorescence occurred from baseline, arguing against a significant contribution from hidden SypHy events. Finally, we also compared events of low and high amplitudes to see if they behave similarly or not (Figure S4), with the idea that multiple simultaneous events, which would most likely occur with the brighter events, would behave differently. However, we saw no difference (Figure S4), arguing against any fundamental difference between small and large events.

Three observations suggest that SypHy fluorescence events predominantly represent the dequenching of a single pHluorin molecule following exocytosis. First, SypHy events occurred infrequently, only in 5.6% of all trials (81 SypHy events in 1,453 line scans). If one assumes a random distribution of SypHy molecules across all ribbons in this study, then the likelihood of two molecules being liberated during a single voltage step would be 0.3% (.056^2^). In 1,453 trials, we expect that this would happen about five times. Second, of the 81 SypHy events, six occurred before (one event) or long after (five events) the voltage step at a time when fusion events are rare (e.g., Figures S3A and 3C) and thus likely reflect a single vesicle. The average amplitude of evoked SypHy events (27.1 ± 1.2, N = 75) is indistinguishable from that for these rare events (25.2 ± 4.9, N = 6) (Figure S3B). Third, the point-spread function (PSF), quantified by the full width at half maximum (FWHM), of a diffraction-limited 27-nm fluorescence bead (Figure S3C) matches the FWHM of SypHy fluorescence obtained in line scans imaged under the same imaging parameters (Figure S3D).

**Figure 3:**
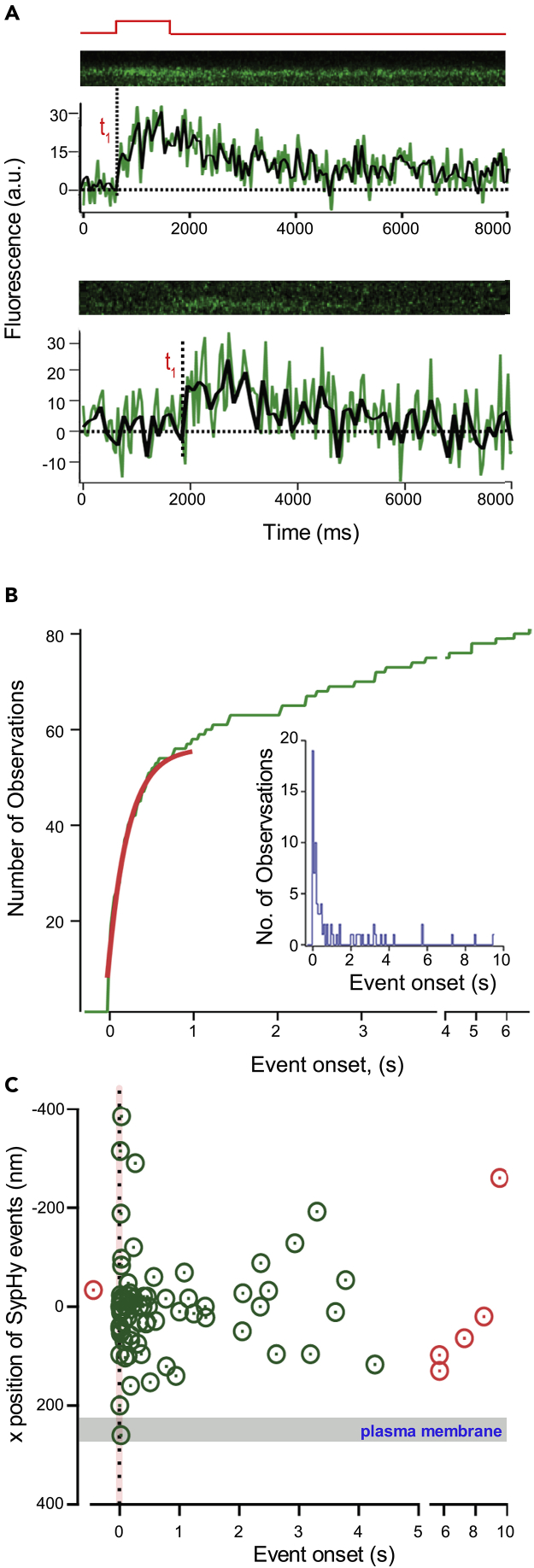
SypHy Events Report Two Components of Vesicle Fusion (A) Examples of SypHy events occurring at different times following depolarization. Event onset (dashed lines, t_1_) was the time when the SypHy fluorescence reached 10% of the event amplitude. (Upper trace) SypHy event onset (1–2 ms) just after the start of depolarization. (Lower trace) SypHy event onset occurred around 1,220 ms after the start of depolarization. The voltage protocol shown at the top (red) is for both recordings. (B) Cumulative distribution of SypHy event onsets (green) up to 5 s after the start of depolarization (N = 75). The red line is a double exponential fitted to time constants of the fast to slow component of capacitance measurements for zebrafish retinal bipolar cells, τ_fast_ = 2.3 ms and τ_slow_ = 162 ms (Mennerick and Matthews, 1996, Vaithianathan and Matthews, 2014, von Gersdorff and Matthews, 1994). The resulting ratio of the fast to slow component of capacitance measurements (1:6) is similar to that obtained for zebrafish bipolar cells (1:5). (Inset) Histogram of the event onset of the 81 SypHy events. (C) x axis position of SypHy events with respect to ribbon PSF (Figure S2) plotted against their event onset. The location of plasma membrane (gray shading) and centroid (x axis) position of SypHy was estimated using sigmoid-Gauss function (see Methods; Figure 1C). Early events, those occurring within 30 ms of depolarization (red shading), occurred both proximal and distal to the plasma membrane (Vaithianathan et al., 2016). SypHy events that occurred up to 4 s after the end of the voltage step (shown in green) were defined as evoked events, whereas SypHy events that occurred before the stimulus or more than 4 s after the end of voltage step are classified as rare (show in red, N = 6).

Because we relate the rate of decline detected in temporal aspects of SypHy fluorescence (Figure 1D, lower panel) to movement of the SypHy molecule from fusion sites, one concern could be stability in tracking the SypHy fluorescence due to drift and photobleaching. However, bleaching rate was minimal during the time course of a typical experiment and accounts for less than 17% with a time constant of 1.8 ± 0.2 s (N = 3) evaluated from line scans obtained from immobilized GFP under the same imaging parameters (data not shown). On average ribeye-binding peptide (RBP) fluorescence was stable over time during an imaging session (data not shown).

### Rate of SypHy Events Occurrence Correlates to Kinetic Components of Bipolar Cell Ribbon Synapses' Exocytosis

Bipolar cell ribbon synapses exhibit two kinetically distinct components of transmitter release in response to step depolarizations: a phasic component that consists of a limited pool of vesicles that can be tapped rapidly but is also rapidly exhausted, generating a transient spike of release, and a tonic component that comprises a much larger pool of more slowly released vesicles (Mennerick and Matthews, 1996, Vaithianathan and Matthews, 2014, von Gersdorff and Matthews, 1994).

The majority of SypHy events occurred soon after the start of membrane depolarization (Figure 3A, upper trace), whereas other events occurred after a delay (Figure 3A, lower trace). To test whether the timing of SypHy events agreed with the known time course of bipolar ribbon synapse transmitter release, we determined the time of onset of SypHy events, relative to the start of membrane depolarization, and plotted them as a cumulative distribution (Figure 3B) and frequency histogram (Figure 3B, inset). We fit the cumulative histogram of event onsets with known kinetic components of release from capacitance measurements for zebrafish and goldfish retinal bipolar cells, τ_fast_ = 2.3 ms and τ_slow_ = 162 ms (Mennerick and Matthews, 1996, Vaithianathan and Matthews, 2014, von Gersdorff and Matthews, 1994). The resulting amplitude-ratio of the fast to slow component of capacitance measurements (1:6) is comparable with measurements obtained for bipolar cells (1:5). These results are consistent with individual SypHy events representing fusion of single vesicles.

We next compared the time of vesicle fusion relative to its location on the ribbon (Figure 3C). Our results show that during the burst phase of vesicle fusion that occurs within 30 ms after the onset of stimulation (Figure 3C, red shading) most events were in the half of the ribbon that was proximal to the plasma membrane (Figure 3C, gray shading). However, there were events also localized distal to the plasma membrane (Figure 3C, negative values). Similarly, SypHy events that occurred after 500 ms of onset of stimulation also had fusion events both proximal and distal to the plasma membrane. These results infer that during sustained stimulation there is no obvious relationship between the timing of events and their location.

### Dynamics of SypHy Fusion Events at a Single Ribbon

**Figure 4:**
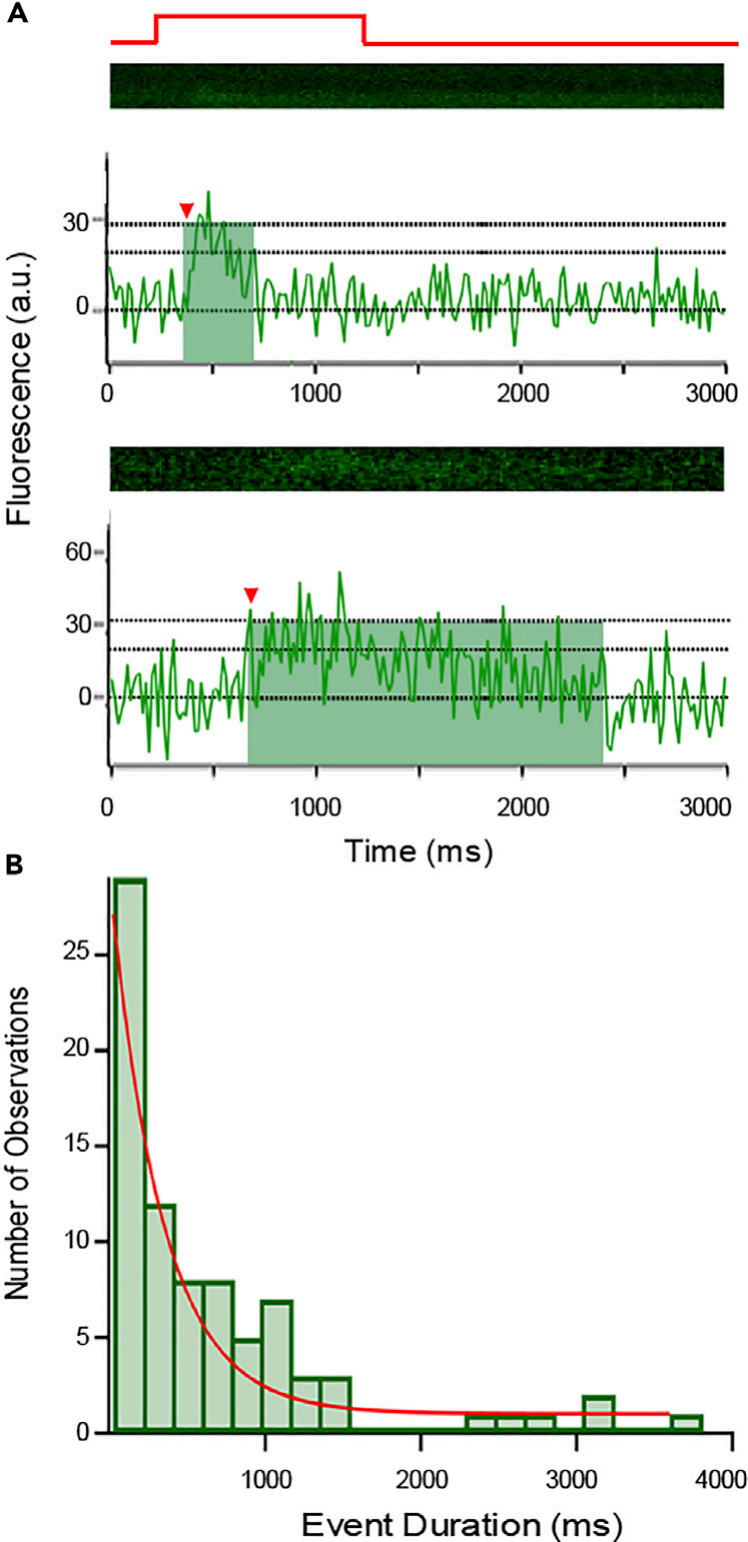
SypHy Fluorescence Persists at the Active Zone after Fusion (A) Examples of x-t scans of SypHy events illustrating variations in the duration of SypHy fluorescence. In some instances, SypHy events were brief (e.g., upper recording), whereas in other instances they persisted longer (e.g., lower recording). To quantify SypHy dwell time or event duration, we measured from event onset (red arrow) to when fluorescence decayed to 1σ (68.3%) of the event amplitude (green shades) (see Methods). Temporal intensity profile is the same as in Figure 1D. We assume the event duration reflects how long the SypHy molecule lingers within the line scan. (B) Histogram of event durations (N = 81 SypHy events) is approximately exponentially distributed, with a time constant of 340 ms.

**Figure 5:**
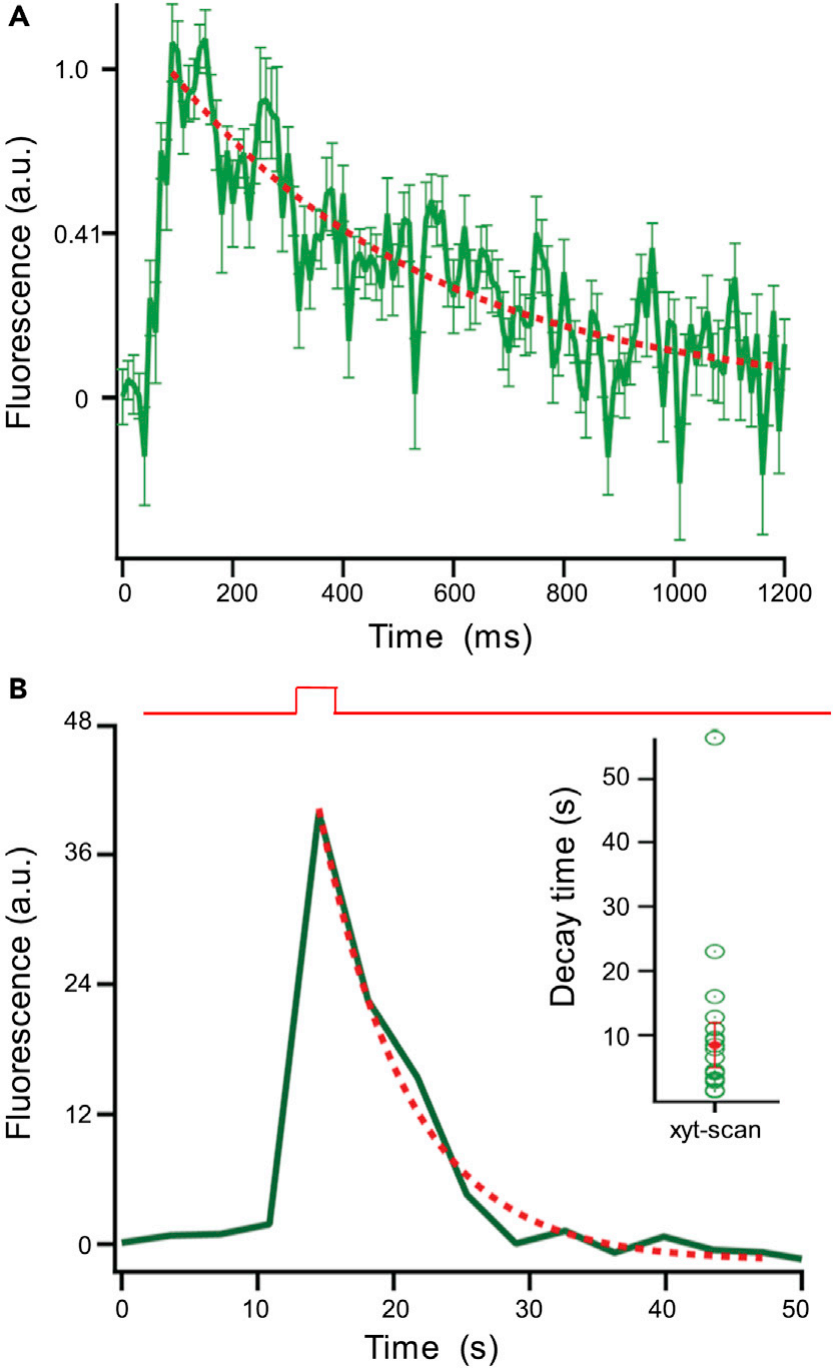
High-Resolution Imaging of a Single SypHy Molecule Reports a Fast Decay Component Attributed to Lateral Movement of Fused Vesicle Protein (A) Ensemble SypHy events (N = 81) from x-t line scans. x-t scans sampled at different rates were interpolated to a common time base (10-ms sample interval) using the interpolation function in Igor. Scans were aligned to event onset and normalized to their event amplitude. The decay phase of the average SypHy event was well fit with an exponential function with τ = 470.7 ± 99.2 ms (N = 81). (B) Example of SypHy fluorescence from an x-y scan taken from a bipolar terminal from highly expressing SypHy fish with a maximally expanded pinhole to assay fluorescence throughout the terminal. Voltage step is shown above. The recovery phase, which presumably reflects the time for internalization, acidification, and photobleaching, was exponential with τ = 4.3 s. In x-y scans (inset) of a square region of interest of 1 × 1 μm centered over a single terminal, recovery of SypHy florescence occurs with a median of 15.0 ± 3.5 s (N = 27 recordings from 12 different terminals).

SypHy signals increased abruptly but remained elevated for some time before returning to baseline (Figure 4). To quantify this SypHy dwell time or event duration, we measured from event onset (red arrow) to when fluorescence decayed to 1σ (68.3%) of the event amplitude (shaded green, Figure 4A) (see Methods). Dwell times were approximately exponentially distributed (Figure 4B) with a time constant of 340 ms. The fluorescence decay of individual SypHy events varied considerably, as expected for the behavior of individual molecules acting stochastically. To analyze the properties of SypHy clearance from the line scan, we averaged SypHy events (N = 81) aligned to the start of event onset (Figure 5A) (see Methods). The decay phase of the averaged SypHy events was roughly exponential with τ = 470.7 ± 99.2 ms. The decay in fluorescence may represent diffusion out of the line scan, photobleaching, or internalization and reacidification. However, the contribution of photobleaching (1.8 ± 0.2 s; 17%) is 5-fold slower than the decay rate (see Methods).

To estimate the possible contribution of endocytosis and reacidification of SypHy molecules to the decay of the signal, we looked at SypHy fluorescence across the entire synaptic terminal with a maximally expanded pinhole to assay fluorescence throughout the terminal (Figure 5B). For these experiments we used a highly expressing SypHy fish (see Methods). By visualizing the entire surface-exposed pool, diffusion should not contribute to the decay in fluorescence, but instead reflects a combination of internalization, acidification, and photobleaching. The decay in the SypHy signals was well fit by a single exponential (Figure 5B, red line). Experiments done on individual ribbons showed a decay time of 15.0 ± 3.5 s (N = 27 SypHy) (Figure 5B, inset). Given that this is much slower than the line scan decay time (about 470 ms) (Figure 5A), this suggests that the fluorescent decay of the individual SypHy events predominantly reflects diffusion out of the small line scan region rather than internalization or photobleaching. Hence, we interpret the individual events as exocytosis liberating single SypHy molecules, followed by diffusion of the fluorescent molecule out of the line scan, where it gradually becomes less fluorescent as it moves away.

### Dynamics of SypHy Clearance

To begin to address the dynamics of clearance from ribbon synapse active zones, we initially considered timing of fusion events. Bipolar terminals show two kinetically distinct components, a fast-transient phase at the beginning of depolarization and a slow-sustained phase occurring at later times. Vesicle clearance during these phases might be quite different owing to the near-simultaneous insertion of significant amounts of vesicle membrane into a confined space, which could either push membrane out of the synaptic region or crowd the active zone plasma membrane with vesicle components.

**Figure 6:**
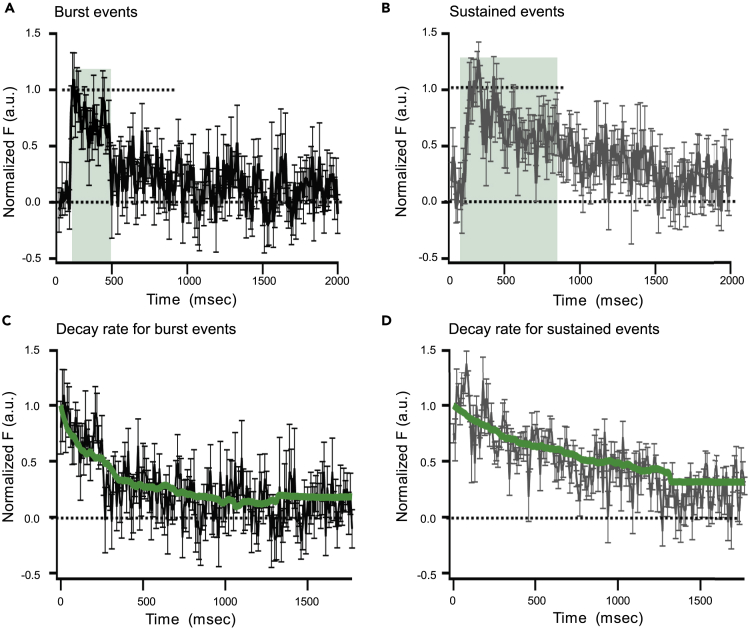
Kinetics of SypHy Events Occurring Early or Late Following the Onset of Depolarization (A and B) Ensemble of SypHy events occurring within 30 ms (A, N = 16, referred to as “burst” events) or after 0.5 s (B, N = 57, referred to as “sustained” events) after the onset of depolarization. SypHy events occurring early in the depolarization or burst events (A) appeared abruptly, remained at a constant level (∼300 ms, green shading), and then declined. SypHy events occurring late in depolarization or sustained events (B) persist longer compared with burst events (∼700 ms, green shading), before decaying. For comparison, fluorescence was normalized to the peak for the individual record (see Methods). (C and D) Decay of ensemble SypHy fluorescence for burst (black, C) or sustained (gray, D) events. We performed Monte Carlo simulations (2D random walk) of diffusional clearance of SypHy from the active zone to compare clearance rates for burst or sustained events (see Methods). The diffusion coefficient was varied to obtain a best fit to the data (green lines). Burst events were best fit by a diffusion coefficient of 41.8 nm^2^/ms, whereas sustained events were best fit with a diffusion coefficient of 9.6 nm^2^/ms.

To define the impact of timing on vesicle clearance, we compared time courses for ensemble averages of SypHy events occurring within 30 ms or 0.5 s after onset of depolarization (Figure 6) (see Methods). We refer to these components as burst (30 ms) or sustained-phase (0.5 s after) events. We found that the dwell time of the ensemble averages was briefer for burst events compared with sustained-phase events (Figures 6A and 6B, green shading) (300 ms versus 700 ms).

To test whether diffusion could explain the decay rate for ensemble SypHy events, we used a Monte Carlo model (see Methods). Briefly, we simulated individual molecules undergoing a random walk in two dimensions, both perpendicular to the axis of the ribbon, one in the z axis and one in the y axis. The fluorescence intensity of the molecule was determined by its distance from the center of the axis of the line scan convolved by the PSF of the microscope, measured with a sub-diffraction limited bead, taking into account the differences in PSF in the z plane and the x/y plane. The only free parameter in the simulation was the diffusion coefficient of the simulated molecules (i.e., step size), which was allowed to vary to best fit the data. For each condition, 84 single molecules were simulated. The diffusion coefficient was varied to obtain a best fit to the data (green lines, Figures 6C and 6D). Burst events were best fit by a diffusion coefficient of 41.8 nm^2^/ms, whereas sustained events were best fit with a diffusion coefficient of 9.6 nm^2^/ms. Thus, during burst events SypHy molecules diffuse out of the active zone more rapidly than during periods of sustained release, an effect possibly related to the total amount of membrane added.

### Clearance of SypHy Molecules Depends upon the Location of Fusion

**Figure 7:**
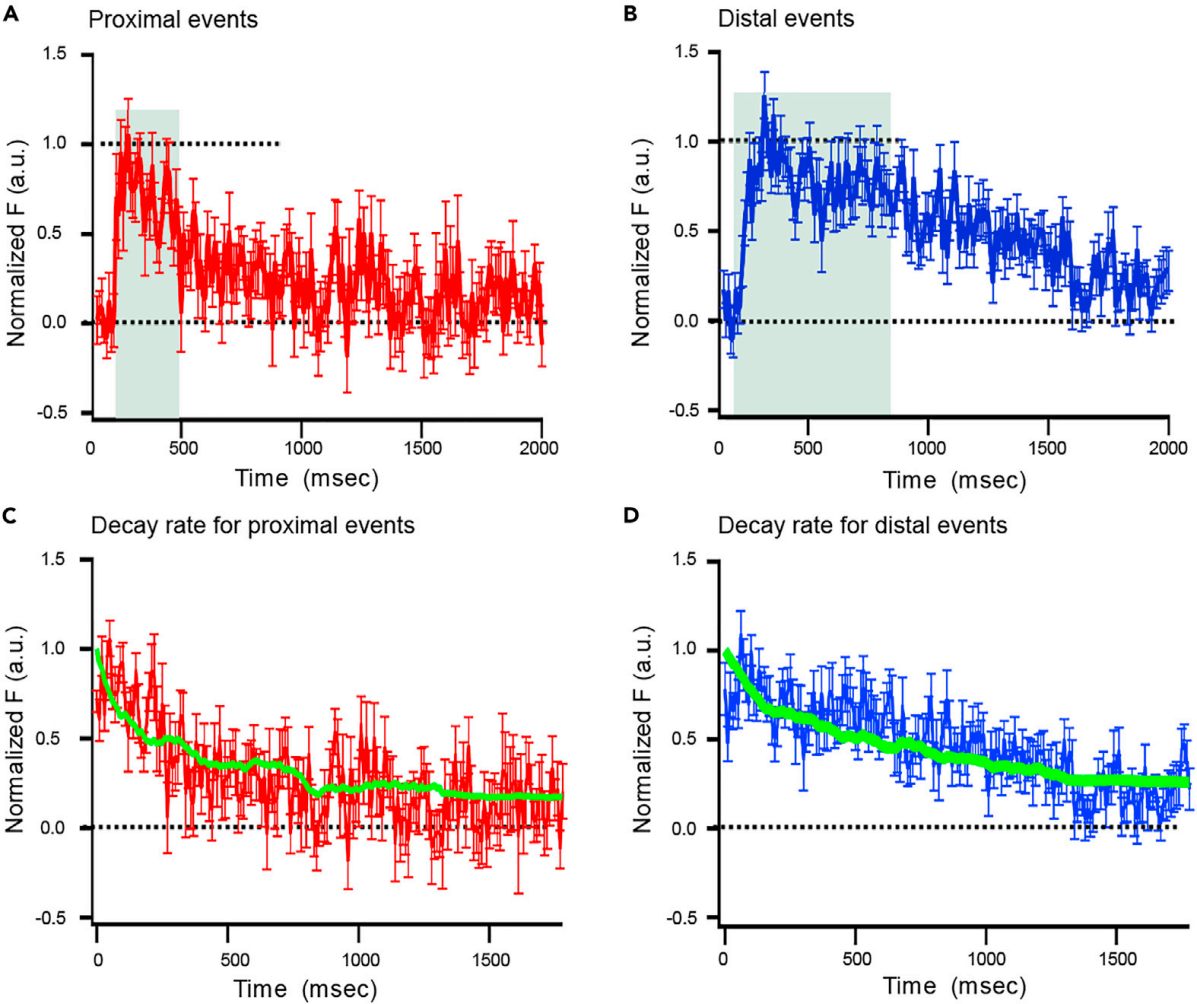
Kinetics of SypHy Events Occurring at Membrane Proximal or Distal Locations (A and B) Ensemble of SypHy events occurring near the membrane, i.e., the x axis position of SypHy events occurring with respect to ribbon PSF ≤ +50 nm (A, N = 25, referred to as “proximal” events) or PSF ≥ −50 nm (B, N = 22, referred to as “distal” events). SypHy events occurring at proximal locations (A) appeared abruptly, remained at a constant level (∼300 ms, green shading), and then declined. SypHy events occurring at distal locations (B) persisted longer compared with burst events (∼700 ms, green shading), before decaying. For comparison, fluorescence was normalized to the peak for the individual record (See Methods). (C and D) Decay of ensemble SypHy fluorescence for proximal (red) or distal (blue) events. Green line indicates best-fit Monte Carlo simulation with a diffusion coefficient of 49 nm^2^/ms for proximal events (C) and 16.1 nm^2^/ms for distal events (D). See also Figure S5.

Vesicle fusion occurred at different locations relative to the plasma membrane (Figure 7). To test whether the location of the fusion event influenced the rate of clearance from the active zone, we grouped SypHy events based on the location relative to the center of the ribbon in the x dimension (x_0_ defined above); SypHy events that occurred at x_0_ > 50 nm and x_0_ < −50 nm were classified as membrane proximal and distal events, respectively. Proximal SypHy events show faster kinetics compared with distal events, where dwell times of proximal events were twice faster than those of the distal events (Figures 7A and 7B) (∼300 ms versus > 700 ms) and the apparent diffusion coefficient estimated with best-fit Monte Carlo simulation resulted in a diffusion coefficient of 49 nm^2^/ms for proximal events and 16.1 nm^2^/ms for distal events.

### SypHy Diffusion at Extrasynaptic Locations

The experiments above addressed movement of recently released SypHy molecules in the restricted area of the synapse. A key question is whether SypHy mobility is different in this restricted environment than in the rest of the synaptic terminal. To address this question, we performed fluorescence recovery after photobleaching (FRAP) experiments in zebrafish bipolar cells that express higher levels of SypHy in retinal bipolar cells (Odermatt et al., 2012). To do so, we measured recovery from photobleaching in a 1 × 1-μm region on the surface of bipolar cell terminals. Photobleaching resulted in a rapid drop in fluorescence, with a recovery that returned to baseline fluorescence over minutes. To extract diffusion coefficient information, we fit to the previously derived equation for 2D diffusion (Axelrod et al., 1976, Feder et al., 1996) (Figure S5):

F(t) = [F_0_ + (R(F^0^ – F_0_)+F_0_)(t/τ)]/(1 + t/τ)

where R represents the fraction of the mobile pool, τ is the half-time of recovery, F^0^ is the fluorescence before bleaching, and F_0_ is the fluorescence immediately after bleaching, and obtained best-fit values of R =.95 and τ = 5578 ms. The diffusion coefficient is determined from the equation (Gomez-Varela et al., 2010):

D = Area of bleach/4τ

yielding an estimate of 44 nm^2^/ms for D. As the vast majority of the terminal is outside the limited active zone, this primarily represents diffusion in extrasynaptic regions of the terminal.

## Discussion

Clearance of synaptic vesicle components following exocytosis from release sites is an important determinant of recovery from short-term synaptic depression, and the clearance and availability of release sites has been suggested to be rate limiting during sustained synaptic release (Hosoi et al., 2009, Hua et al., 2013, Wen et al., 2018, Wu et al., 2009). At the rod ribbon synapse, pharmacological blockage of dynamin-dependent endocytosis strongly reduces subsequent exocytosis without changing vesicle docking rates, suggesting that release site clearance is necessary to maintain release at these synapses (Wen et al., 2018). Nevertheless, little information has been available about the dynamics of newly fused vesicle proteins at the active zone. In the present study, we directly addressed this question using single-molecule imaging at the active zone of a living neuron and derived real-time information about the clearance of individual synaptic vesicle proteins following exocytosis. To study these processes, we monitored the fate of synaptophysin molecules following exocytosis from zebrafish bipolar cell ribbon synapses. Our results demonstrate that the clearance of synaptophysin from the synapse is influenced by the time and location of vesicle fusion and suggest a model that supports clearance of massive amount of membrane added at the beginning of a voltage step.

Synaptophysin is a synaptic vesicle protein that regulates endocytosis of synaptic vesicles during and after sustained transmission (Kwon and Chapman, 2011). Synaptophysin has also been suggested to act as a release site clearance factor, preventing synaptobrevin 2 forming *cis*-SNARE complexes from obstructing release sites (Rajappa et al., 2016). To track newly released synaptophysin, we capitalized on our ability to localize active zones using a ribbon-binding peptide (Zenisek et al., 2004). Our results differ from previous measurements of mobility of membrane proteins using single particle tracking, fluorescence recovery after photobleaching, and fluorescence correlation spectroscopy in that our approach is able to distinguish newly released protein following exocytosis. Protein diffusion is influenced by interplay of protein-to-lipid ratios (Ramadurai et al., 2009), curvature of the membrane (Leitenberger et al., 2008), membrane tension (Quemeneur et al., 2014), lipid packing (Javanainen et al., 2013), and protein shape. To our knowledge, only Gimber et al. (2015) have combined tracking newly exocytosed synaptic vesicle proteins with localizing active zone in neurons, and then only in sequentially acquired images separated by some time, which were intended to test whether observed newly exocytosed pHluorin coincided with active zone marker, Munc 13-1. By using two-color laser scanning confocal microscopy, we were able to detect the locus of fusion and track individual SypHy molecules following exocytosis during sustained depolarization using live cell imaging in voltage-clamped synaptic terminals.

Our results demonstrate that the rate of fluorescence decline and thus SypHy clearance is influenced by the timing and location of vesicle fusion at bipolar cell ribbon synapses. Our results indicate a faster apparent diffusion coefficient for burst events (41.8 nm^2^/ms) compared with late events (9.6 nm^2^/ms). It should be noted that this difference may be an underestimate, because longer dwell times would be expected to be contaminated to a greater degree by photobleaching. These results suggest that the burst phase of exocytosis enhances clearance from the synapse. To account for this difference, we suggest a model in which the massive amount of membrane added at the beginning of a voltage step may act to push synaptophysin and other synaptic vesicle components from the synapse. The transient component of zebrafish bipolar cells measured by capacitance measurements suggests that about 1,200 synaptic vesicles are released with a time constant of 2.3 ms (Vaithianathan and Matthews, 2014) at approximately 50 ribbon sites, an average of 24 per ribbon. On average, this burst phase would be expected to add 0.07 μm^2^ of membrane per active zone assuming a vesicle diameter of 30 nm. The synaptic ribbon at the base covers a roughly rectangular region of membrane 0.4 μm long by 0.15 μm wide for an area of 0.06 μm^2^ (von Gersdorff and Matthews, 1996, von Gersdorff and Matthews, 1999). Hence, the burst phase of exocytosis is expected to more than double the active zone membrane in <5 ms. Ca^2+^ channels at photoreceptor synapses also exhibit increased mobility at times of high rates of exocytosis (Mercer et al., 2011), suggesting that this may be a general feature of ribbon synapses. Interestingly, unlike SypHy, Ca^2+^ channels in both bipolar cells and photoreceptors appear to be confined to a restricted region, which can be disrupted by reagents that disrupt the actin cytoskeleton (Mercer et al., 2011, Thoreson et al., 2013).

Release site clearance is likely a complex process and may be influenced by multiple factors (Haucke et al., 2011, Neher, 2010). Previous measurements of diffusion coefficients of membrane proteins have ranged from 1–300 nm^2^/ms (Bannai et al., 2009, Dahan et al., 2003, Funahashi et al., 2018, Gomez-Varela et al., 2010, Groc et al., 2008, Mercer et al., 2011, Mikasova et al., 2008), placing our values of ∼42 nm^2^/ms during the burst phase and ∼10 nm^2^/ms during the sustained phase at the low end of membrane protein measurements and much lower than the previous report on synaptophysin in hippocampal synapses (180 nm^2^/ms) (Funahashi et al., 2018). We propose the following three hypotheses for why our results may differ: (1) the ribbon synapse may be more densely packed with protein and other diffusion barriers than a conventional synapse or perisynaptic regions; (2) our cells exhibit minimally expressing synaptophysin-pHluorin molecules, whereas previous measurements were maximized for visualizing release and hence exhibited a much higher concentration of released synaptophysin; if fixed synaptophysin-binding proteins reside within the active zone, binding sites may be saturated in their model allowing for free diffusion; and (3) synaptophysin may have different mobility in the membrane of fish bipolar cells than in mouse hippocampal neurons. To begin to address this issue, we used FRAP to look at the diffusion coefficient of SypHy in a different line of zebrafish with higher expression on the cell surface. Because the region immediately below the ribbon is a small fraction of the total area of the terminal, these measurements are dominated by SypHy in extrasynaptic regions. With the FRAP data, we find that the diffusion coefficient in the extrasynaptic membrane is 44 nm^2^/ms, similar to burst phase around the ribbon and about 4-fold faster than when events are rare, but still much slower than results in hippocampal neurons. These results suggest that the region around the ribbon may exhibit lower protein mobility at rest than the surrounding plasma membrane. Based on these results, we suggest that the region immediately surrounding the synapse may be a region that contains a higher density of diffusion barriers that may restrict movement of molecules, but that the membrane flow associated with exocytosis may be able to overcome this restricted mobility. In addition, our results show that synaptophysin movement is more restricted in the bipolar cell than in hippocampal neurons and that transgene expression level is unlikely to account for our differences. Together our results suggest that plasma membrane structure may vary between these different types of cells and regions, because lateral diffusion is highly sensitive to protein-lipid contacts (Knight et al., 2010) and protein concentration (Goose and Sansom, 2013) and protein-to-lipid ratio varies extensively across different microstructures of biological membrane (Dupuy and Engelman, 2008).

Interestingly, studies from photoreceptor synapses (Mercer et al., 2011) and bipolar cells (Thoreson et al., 2013) found that the α2δ subunit of voltage-gated Ca^2+^ (Ca_V_) channels positioned close to the synaptic ribbons exhibited diffusion within a restricted domain, but were confined to a location surrounding the active zone. Despite being embedded within a much larger complex than synaptophysin, α2δ subunits exhibited diffusion coefficients within the restricted domain of 170 nm^2^/ms in photoreceptors and 300–450 nm^2^/ms in bipolar cells, an order of magnitude higher than our measurements. The calcium channel measurements were accomplished by coupling α2δ subunits to quantum dots via antibodies. The superior fluorescent properties of quantum dots enable the authors to track smaller lateral movements with much higher spatial resolution than we are able to achieve with single-molecule imaging of GFP. Hence, it is possible that synaptophysin undergoes similar rapid diffusion on a local scale that goes undetected with our techniques.

Our results indicate that the dynamics of clearance is accelerated at times when exocytosis rates are high or when vesicles fuse in proximity to the plasma membrane. However, it is not clear whether the proximity of the vesicles to the plasma membrane or the overall exocytosis rate is the dominant factor in determining clearance rates. This poses an interesting avenue for future research using our approach. The dynamics of release site clearance was argued as a limiting and modulatory element in synaptic transmission. Further studies are required to determine the mode of vesicle fusion and limits of bipolar cell ribbon synapse to sustain the transmission during strong stimulation for encoding background illumination. We believe that our direct approach of imaging single vesicles (Vaithianathan et al., 2016) in conjunction with capacitance measurements and tracking clearance of fused vesicle protein demonstrated in this study are valuable tools that can be combined in the future to explore this question.

### Limitations of the Study

The limitation of the study is governed by optimizing parameters to track a single molecule at a single active zone in a living terminal for several seconds. The ability to obtain such measurements is limited by several factors: the optics, health of the neuron, incorporation of the transgene, the stability of the system, the trade-off between signal-to-noise ratio (SNR), and bleaching. We acknowledge that our study could have failed to detect smaller lateral movements due to SNR, attributes to rapid diffusion demonstrated previously for other synaptic proteins.

## Methods

All methods can be found in the accompanying Transparent Methods supplemental file.

## Supplemental Information

Document S1. Transparent Methods and Figures S1–S5
