# Supplementary material for: Tracking Newly Released Synaptic Vesicle Proteins at Ribbon Active Zones
Source: iScience. 2019 Jun 13;17:10–23. doi: 10.1016/j.isci.2019.06.015 (PMC6598641; doi:10.1016/j.isci.2019.06.015)
Supplement: Document S1. Transparent Methods and Figures S1–S5 [file mmc1.pdf]

ISCI, Volume *17*

Supplemental Information

Tracking Newly Released Synaptic Vesicle Proteins at Ribbon Active Zones

Thirumalini Vaithianathan, Lonnie P. Wollmuth, Diane Henry, David Zenisek, and Gary Matthews

Supplemental materials

Transparent Methods

Transgenic animals

Sparse expressing adult HSP70::SypHy Zebrafish (Vaithianathan et al., 2016) of both sexes were incubated in water bath at 37°C for 2 hours one day prior to experiments to drive protein expression via the HSP70 promoter. A line of fish generated in the same manner had higher expression and were used for the experiment in Figure 5B. Animals were maintained according to NIH guidelines and all procedures were approved by the Institutional Animal Care and Use Committee of the State University of New York at Stony Brook.

For FRAP experiments (Figure S5), a transgenic line of zebrafish, kindly donated by Dr. Leon Lagnado (University of Sussex) that express SypHy off of the Ribeye A promoter (Ribeye::sypHy) were used (Odermatt et al., 2012).

Electrophysiology and analysis

Dissociation of zebrafish retinal bipolar cells: The procedure for isolating bipolar neurons from zebrafish retina was as described previously (Vaithianathan et al., 2013a). Briefly, retinas were dissected from the eyecups, treated for 25 min with hyaluronidase (1100 units/ml), cut into small pieces, and incubated 15-40 min in saline [(in mM) 115 NaCl, 2.5 KCl, 0.5 $CaCl_2$, 1 $MgCl_2$, 10 HEPES, 10 Glucose, pH 7.4], containing DL-cysteine and 15-30 units/ml papain (Sigma) at 25 ºC for 30~40 min. A piece of retina was gently triturated using a fire-polished glass Pasteur pipette and dissociated cells were plated onto glass-bottomed dishes containing a saline solution

(Heidelberger and Matthews, 1992) with $CaCl_2$ increased to 2.5 mM. ON bipolar cells were identified by their characteristic morphology.

Bipolar cell voltage clamp recordings: Whole-cell patch-clamp recordings were made from acutely dissociated bipolar cells by directly placing the patch pipette on the synaptic terminal (Vaithianathan et al., 2013b). The patch pipette solution consisted of (in mM): 120 Cs-gluconate, 10 tetraethylammonium-Cl, 3 $MgCl_2$, 0.2 *N*-methyl-d-glucamine-EGTA, 2 Na_2ATP, 0.5 Na_2GTP, 20 HEPES, pH 7.2. and bath solutions (saline, with 2.5 mM $CaCl_2$). The patch pipette solution included fluorescent RBP peptide (deep-red CF633-RBP) to mark ribbons (Zenisek et al., 2004), and 3 mM reduced glutathione as a free-radical scavenger. Membrane currents were recorded under voltage clamp using a HEKA EPC-9 amplifier controlled by PatchMaster software (HEKA). For all recordings, the holding potential was -60 or -65 mV and stepped to -10 mV (t_0) for 1s. Membrane capacitance, series conductance, and membrane conductance were measured using the sine DC method of the PatchMaster lock-in extension and a 1600 Hz sinusoidal stimulus with peak-to-peak amplitude of 10 mV centered on the holding potential (Vaithianathan et al., 2013b). Pipettes were coated with dental wax to reduce stray capacitance.

Image collection

Confocal images were acquired using Olympus FluoView software controlling an Olympus FV1000/IX-81 laser-scanning confocal microscope equipped with a 60X 1.42 NA oil-immersion objective. For experiments monitoring global SypHy responses to sustained depolarization, x-y raster scans of a zoomed terminal were acquired with scan speed of 2 μs/pixel (frame size, 256 × 256 pixels). For these experiments, bipolar terminals were stimulated at 30-s interval. Terminals with leak current >20 pA were not included in analysis.

For the vast majority of our experiments, we generated rapid x-t line scans of a zoomed region through a selected ribbon to monitor green (SypHy) fluorescence. These x-t line scans were of a duration of 1-8 msecs. To avoid potential errors arising from high curvature near the top of the terminal and the plane of adherence of the membrane to the glass coverslip at the bottom of the terminal, we carefully adjusted the focal plane to bring labeled ribbons into sharp focus. This procedure minimized curvature of the plasma membrane in the z-axis within the optical section and therefore facilitated localization of the plasma membrane with respect to the ribbon. Quantitative image analysis was performed by importing FluoView images of x-y and x-t scans into ImageJ (imagej.nih.gov) for initial processing and analysis. Subsequent analysis was performed in Igor Pro (Wavemetrics, Portland OR) using custom-written routines. Timing between voltage-clamp recording and imaging was synched by generating a horizontal-scan synch pulse by the hardware of the confocal microscope, in parallel with the voltage-clamp data (PatchMaster).

We confirmed for possible bleed-through before conducting experiments by imaging each channel with both lasers in the same imaging parameters used for experiments in bipolar cells isolated from wild-type zebrafish. To test the bleed-through from RBP channel (LD 635) to SypHy Channel (LD 488) we patched wild-type bipolar cells with patch pipette solution included fluorescent RBP peptide (deep-red CF633-RBP) to mark ribbons, performed line-scan images and analyzed in the same manner will be used for experiments. To look for possible bleed through from SypHy channel to RBP channel we followed the same procedure for GFP molecules.

Photobleaching: To minimize photobleaching and phototoxicity during live-cell imaging, we used fast scan speed (2~10 μs/pixel), low laser intensity (0.1–0.5% of maximum), and low pixel

density (frame size, 256 × 256 pixels). Photo bleaching with our imaging parameters were estimated using immobilized GFP. x-t line scans of immobilized GFP using these same imaging parameters.

Image analysis

Analysis of x-y scans: SypHy responses in x-y scans were identified using 'Image Calculator" (imagej.nih.gov) to identify loci of stimulus-dependent increases of SypHy fluorescence across the terminal. To increase the overall intensity and to improve the signal to noise ratio (SNR), we increased the pinhole. Increasing the pinhole extended the z-axis resolution to 10 μm as it collects signal from layers outside the focal plane. Square ROIs (1 x 1 μm) were then positioned at such exocytic hotspots, confirmed by RBP fluorescence, revealing active synapses. Changes in fluorescence were quantified by subtracting a three-frame average obtained immediately before depolarization from a comparable average obtained during the sustained depolarization. The recovery or decay back to baseline phase were fitted to declining exponential.

Analysis of x-t scans: SypHy Zebrafish sparsely express SypHy (Vaithianathan et al., 2016). We recorded a total of 1453 x-t line scans in response to sustained depolarization from different ribbons, and 36 line scans without membrane depolarization (controls). This data set was collected from about 1200 different terminals since in some instances we recorded from different ribbons in the same terminal. To detect a SypHy event, in a x-t line scan, we initially examined the temporal profile of x-t line around the region of interest (ROI) encompassing the ribbon and cell interior (Figure S1). We identified 81 putative SypHy events that demonstrated an increase in green fluorescence over baseline. We imaged 27-nm beads and GFP (kind gift from Dr. Mark Bowen, Stony Brook University) fixed on the glass bottom dish used for experiment

using streptavidin and biotin with the same imaging parameters used to detect SypHy events. To quantify these events, we performed single molecule image analysis:

Single molecule image analysis

x-t scans were performed at rates that varied from 125 to 1,000 Hz. To optimize quantification of RBP and SypHy fluorescence, we averaged x-t images over 2 pixels in the x-axis and 5-8 lines in the t-axis. In some instances, as delineated below, we further averaged the images.

x-axis profile: To determine the location of SypHy events with respect to ribbon location, we fit x-axis intensity profiles with the equation f(x) = s(x) + g(x) (Vaithianathan et al., 2016). Here, s(x) is a sigmoid describing the transition from intracellular to extracellular background fluorescence at the edge of the cell, given by $s(x) = b- (c/(1\text{-}exp((x_{1/2}\text{-}x)/d)))$, and g(x) is a Gaussian representing the fluorescence of RBP and SypHy, given by $g(x) = a(exp(-(x\text{-}x0)^2/w^2))$. The parameters $x_{1/2}$ and x_0 were taken as the x-axis positions of the plasma membrane and the fluorescence emitter, respectively. The parameter b is intracellular background fluorescence, c is extracellular background fluorescence, d is the slope factor of the sigmoid, a is the peak amplitude of emitter fluorescence, and w is √2* the standard deviation of the Gaussian. In practice, the latter parameters were highly constrained by the data or by the measured PSF, essentially leaving only $x_{1/2}$ and x_0 as free parameters in the fitting.

Analysis along t-axis profile

To compare SypHy events obtained from different experiments, we defined several parameters:

Event amplitude: To measure the amplitude of SypHy events, we took the difference between the 'peak' amplitude of the SypHy event and the baseline, the average fluorescence obtained

immediately before depolarization. To measure the ‘peak’ amplitude, we identified the maximum value that occurred early during the SypHy event and averaged 5 points centered around the peak. To define the limits of small amplitude events, we randomly chose 203 out of 1372 null events and measured ‘event amplitudes’ by taking the difference between average fluorescence immediately after the depolarization and the baseline, averaging 5 points.

To evaluate the imaging noise, measurements were obtained using the same approach as with SypHy and nulls except that noise measurements were made in the absence of a stimulus (N=36). The results were normalized to the total number of trials and fit with Gaussian function red (red; σ=4.9.

Point spread function (PSF): To characterize the PSF, we used the full width at half maximum (FWHM) (citation). The lateral and axial point spread function is obtained by taking an XYZ scan through a single 27 nm bead. The maximum projection in the xy-plane was fit to the Gaussian function, $g(x,y) = z\_0 + [(-1)/2(1 - 〖cor〗^2)\ (((x - x\_0)/xwidth)^2 + ((y - y\_0)/ywidth)^2 - 2cor(x - x\_0)(y - y\_0)/(xwidth \cdot ywidth))]$ to obtain the lateral resolution (FWHM). Similarly, the y-z axis projection was fit to the Gaussian function, $g(y,z) = z\_0 + [(-1)/2(1 - 〖cor〗^2)\ (((y - y\_0)/ywidth)^2 + ((z - z\_0)/zwidth)^2 - 2cor(y - y\_0)(z - z\_0)/(ywidth \cdot zwidth))]$ to obtain the axial resolution. FWHMs that we obtained from x-width was 259 nm in the lateral (x-y plane) and y-z-width was 448 nm in the axial (y-z axis) resolution.

To determine the FWHM of SypHy events, we fit x-axis intensity profiles with the equation f(x) = s(x) + g(x) (Vaithianathan et al., 2016). Here, s(x) is a sigmoid describing the transition from intracellular to extracellular background fluorescence at the edge of the cell, given by $s(x) = b- (c/(1-\exp((x_{1/2}-x)/d)))$, and g(x) is a Gaussian representing the fluorescence of

SypHy, given by $g(x) = a(\exp(-(x-x0)^2/w^2))$. The parameters $x_{1/2}$ and x_0 were taken as the x-axis positions of the plasma membrane and the fluorescence emitter, respectively. Here the parameter w (width) is √2* the standard deviation of the Gaussian, which we used to determine the FWHM.

Event onset: To determine the time of SypHy event occurrence, referred to as t_1, we identified the time when the SypHy fluorescence reached 10% of the event amplitude. Due to noisiness of the traces, the 10% value was often difficult to define. In such cases, traces were subjected to additional temporal averaging to estimate event timing.

Dwell time: SypHy events remain at fusion sites with different durations before declining to baseline fluorescence. We refer this period as event duration or dwell time, which we quantified by measuring the duration between the time of SypHy event occurrence (t_1), to fluorescence decline to 1σ (68%) of event amplitude.

Ensemble averages: To categorize SypHy events we grouped the averaged records as following: we initially generated a common time base (10 ms sample interval) by interpolating values using IGOR Pro and aligned to start of event onset. Resulting events were grouped into different categories after normalizing to their event amplitude. To compare SypHy events occurring during burst vs. sustained component of release, we grouped SypHy events occurring within 30 ms from start of depolarization or 500 ms after onset of depolarization.

For locus of vesicle fusion, we grouped SypHy events based on the location relative to the center of the ribbon in the x-dimension (x_0 defined above)***;*** SypHy events that occurred at $x_0 > 50$ nm and $x_0 < -50$ nm were classified as membrane proximal and distal events, respectively.

Simulation of the random-walk of single molecules.

We performed Monte Carlo simulations of a molecule undergoing a 2D random walk illuminated by a line scan. The random-walk was constructed for 84 single-molecule diffusion trajectories using a routine written in Excel. By convolving the location of the molecule to Gaussian function with the spatial (x-axis), and axial (z-axis) PSF of the microscope, we were able to determine the expected change in fluorescence as a function of time for clearance of SypHy molecules by diffusion for various diffusion coefficients (D). A best-fit was determined by varying D to minimize the squared difference between the observed and simulated curves.

Statistical methods

No statistical method was used to predetermine sample size. Variance in estimates of the population mean is reported as ± sem. Statistical significance of differences in average amplitudes of rare and evoked events was assessed using unpaired, two-tailed *t* tests with unequal variance.

Supplemental Figure legends

Figure S1, relates to Figure 1. Analysis of SypHy fluorescence along t-axis.
(A) ***Upper***, Raw x-t raster plot, obtained at 2.36 ms/line consisting of 1026-line scans, showing t-axis intensity profiles of SypHy fluorescence (green) during sustained depolarization (same record as in Figure 1). The ROI, the area between the two white dashed lines, was obtained from the raw x-axis intensity profile (B, green).
Lower, Same line scan as in A but averaged, using the integration function in ImageJ, over 2-pixels along the x-axis and 5 scan lines along the time axis to enhance the SypHy fluorescence signal. The two dashed lines indicate the ROI, obtained in the same manner explain for raw line scan. The spatial profile for averaged line-scan is shown in *A, middle-black* trace.
(B) Spatial (x-axis) intensity profile of x-t raster scan, either the raw (green) or averaged (black) scan in A. The green dash line is centroid (x_0) of the Gaussian fit of the SypHy fluorescence. The black (white lines in A) lines are ± 1 σ of the centroid and were used to define the ROI in upper scan in A. Spatial profiles were obtained as in Figure 1C.
(C) Temporal (t-axis) intensity profile of SypHy fluorescence taken from the ROI either for the raw (green, upper scan in A) or averaged (black, lower scan in A) scan. The scans were similar in overall shape and magnitude, but the averaged scan allowed for more quantitative analysis.

Figure S2, relates to Figure 1: Spatial distribution of SypHy events.
Histogram of the x-axis position of 81 SypHy events during sustained depolarization. The relative position of the plasma membrane (solid arrow) and membrane curvature (dashed arrow) were obtained as described previously (Vaithianathan et al., 2016).

Figure S3, relates to Figure 2. Evidence for single molecule imaging.
(**A**) Example of a SypHy event (arrow) that occurred at a time > 4 s after the end of the depolarizing step (t_1). SypHy events unrelated to the depolarizing step, occurring 4 s after the end or before the depolarizing step, are classified as rare events. A total of 6 SypHy events were classified as rare.
(**B**) Average amplitudes of rare (25.2 ± 4.9, N = 6) and evoked (and 27.1 ± 1.2, N = 75) events. Values are not statistically different (*t-test*, p <0.05).
(**C**) The PSF of a 27-nm fluorescence bead (black). The PSF was obtained from the fit with a Gaussian function and were quantified using the full width at half maximum (FWHM) (see Materials & Methods).
(**D**) ***Left,*** Spatial profile comparison of a single 27-nm bead (C) with SypHy fluorescence (D) derived from x-t line scans. One-dimensional Gaussian fitted to x-t line scans of a single 27-nm bead or Sigmoid-Gaussian fitted SypHy fluorescence by using Igor Pro. The indicated width (0.14 ± 0.2 µm, N = randomly chose 3 out of 81 SypHy events) of the spatial profile of SypHy fluorescence obtained from the Sigmoid-Gaussian fit (see Materials & Methods; Figure 1C) matches the width of a single 27-nm bead (0.12 ± 0.2 µm, N = 4).
Right, Scatter plot of spatial profile of SypHy fluorescence obtained from the Sigmoid-Gaussian fit. Average width (blue diamond), 0.14 ± 0.03 µm; N = 40. Note, we limited this analysis to only the brighter SypHy events, since those would be the most likely to arise from multiple molecules.

Figure S4, relates to Figure 2. Decay kinetics are independent of event amplitude.
(**A**) Average of SypHy events with amplitudes above 30 a.u. (A, black line; N = 36) or below 20 a.u. (A, gray line; N = 33). Scans were aligned to event onset and normalized to their event amplitude.
(**B**) Scatter plot of event amplitude (pA) vs. dwell time (ms) of all events (N = 81). The dash line (blue) show the best fit (r = −0.3)

Figure S5, relates to Figure 7. Fluorescence recovery after photobleaching. Average fluorescence from 18 1 µm x 1 µm square region showing recovery after being subjected to intense light (21.6 ms) to induce photobleaching. Red line represents best-fit to a 2D diffusion model (Axelrod et al., 1976).

Supplemental Figures

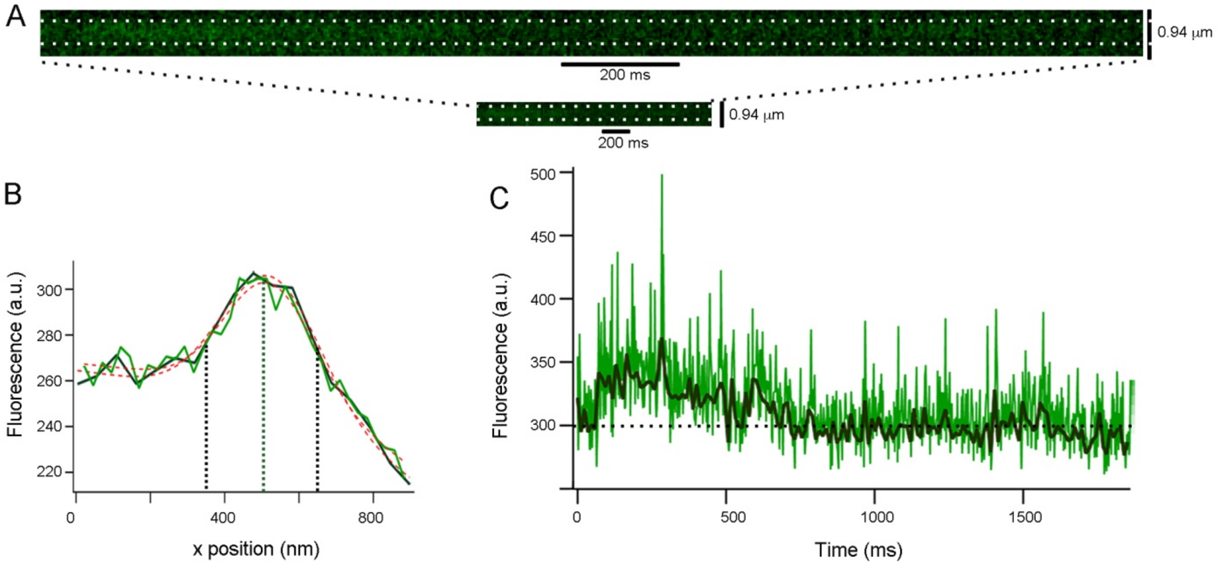

Figure S1, relates to Figure 1. Analysis of SypHy fluorescence along t-axis.

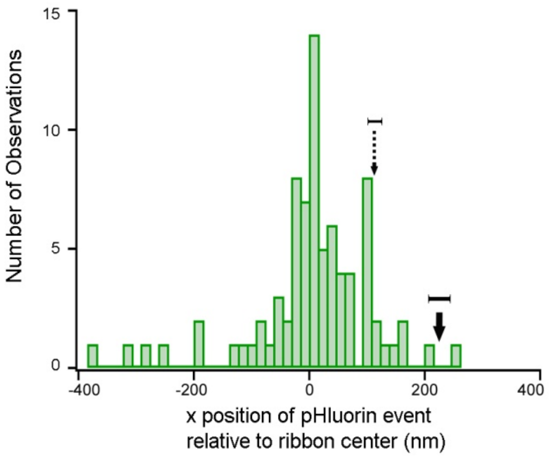

Figure S2, relates to Figure 1: Spatial distribution of SypHy events.

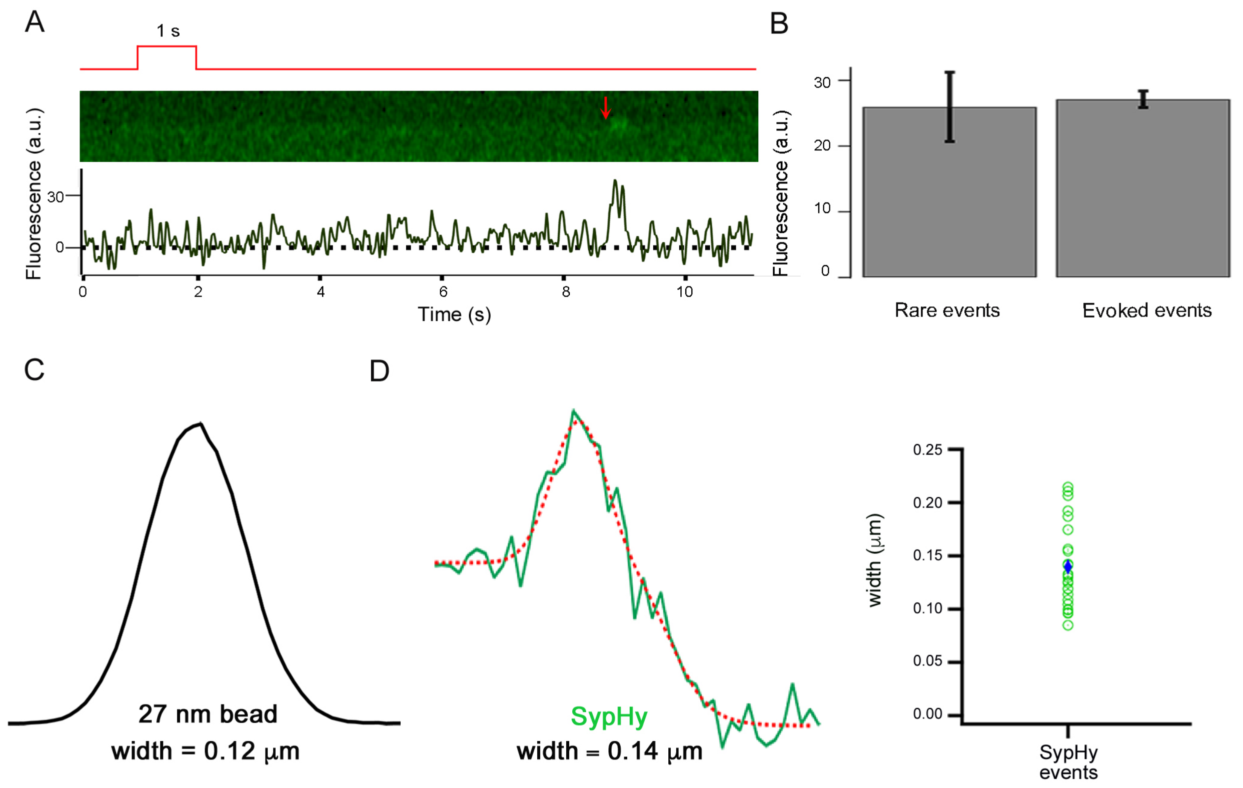

Figure S3, relates to Figure 2. Evidence for single molecule imaging.

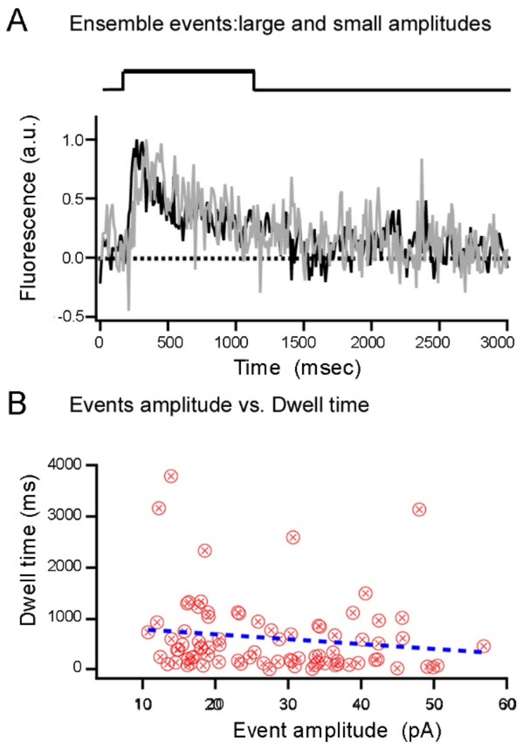

Figure S4, relates to Figure 2. Decay kinetics are independent of event amplitude.

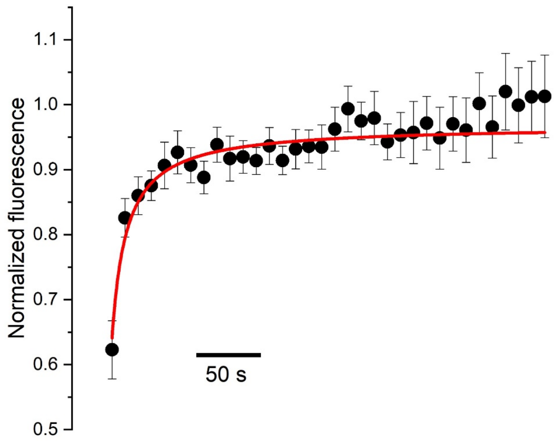

Figure S5, relates to Figure 7. Fluorescence recovery after photobleaching.

Supplemental References

Axelrod, D., Koppel, D.E., Schlessinger, J., Elson, E., and Webb, W.W. (1976). Mobility measurement by analysis of fluorescence photobleaching recovery kinetics. Biophys J *16*, 1055-1069.

Heidelberger, R., and Matthews, G. (1992). Calcium influx and calcium current in single synaptic terminals of goldfish retinal bipolar neurons. J Physiol *447*, 235-256.

Odermatt, B., Nikolaev, A., and Lagnado, L. (2012). Encoding of luminance and contrast by linear and nonlinear synapses in the retina. Neuron *73*, 758-773.

Vaithianathan, T., Akmentin, W., Henry, D., and Matthews, G. (2013a). The ribbon-associated protein C-terminal-binding protein 1 is not essential for the structure and function of retinal ribbon synapses. Mol Vis *19*, 917-926.

Vaithianathan, T., Henry, D., Akmentin, W., and Matthews, G. (2016). Nanoscale dynamics of synaptic vesicle trafficking and fusion at the presynaptic active zone. Elife *5*.

Vaithianathan, T., Zanazzi, G., Henry, D., Akmentin, W., and Matthews, G. (2013b). Stabilization of spontaneous neurotransmitter release at ribbon synapses by ribbon-specific subtypes of complexin. J Neurosci *33*, 8216-8226.

Zenisek, D., Horst, N.K., Merrifield, C., Sterling, P., and Matthews, G. (2004). Visualizing synaptic ribbons in the living cell. J Neurosci *24*, 9752-9759.
